# Educational needs in patients with spondyloarthritis in Sweden – a mixed-methods study

**DOI:** 10.1186/s12891-017-1689-8

**Published:** 2017-08-02

**Authors:** Emma Haglund, Ann Bremander, Stefan Bergman, Ingrid Larsson

**Affiliations:** 1 School of Business, Engineering and Science, Halmstad University, Halmstad, Sweden; 2 Spenshult Research and Development Center, Bäckagårdsvägen 47, SE-30274 Halmstad, Sweden; 3 Department of Clinical Sciences, Lund, Section of Rheumatology, Lund University, Lund, Sweden; 4 Primary Health Care Unit, Department of Public Health and Community Medicine, Sahlgrenska Academy, University of Gothenburg, Gothenburg, Sweden; 5 School of Health and Welfare, Halmstad University, Halmstad, Sweden

**Keywords:** Patient education, Spondyloarthritis, Ankylosing spondylitis, Undifferentiated spondyloarthritis, Patient-reported outcome measures, Interviews

## Abstract

**Background:**

There is a demand for a flexible and individually tailored patient education to meet patients’ specific needs and priorities, but this area has seldom been studied in patients with spondyloarthritis (SpA), a family of inflammatory rheumatic diseases. The aim of the present study was to identify needs and priorities in patient education in patients with SpA. A second aim was to investigate patients’ experiences and preferences of receiving patient education.

**Methods:**

Data collection included a questionnaire survey with the Educational Needs Assessment Tool (ENAT) and interviews, using a mixed-methods design. Patients were identified through a specialist clinic register. Descriptive data are presented as mean with standard deviation, or frequencies. Chi-square test and independent-samples t-test were used for group comparisons. A manifest qualitative conventional content analysis was conducted to explore patients’ experiences and needs in patient education, based on two focus groups (*n* = 6) and five individual interviews.

**Results:**

Almost half (43%) of the 183 SpA patients had educational needs, particularly regarding aspects of self-help, feelings, and the disease process. More educational needs were reported by women and in patients with higher disease activity, while duration of disease did not affect the needs. The qualitative analysis highlighted the importance of obtaining a guiding, reliable, and easily available patient education for management of SpA. Individual contacts with healthcare professionals were of importance, but newer media were also requested.

**Conclusion:**

There are considerable educational needs in patients with SpA, and education concerning self-help, feelings, and the diseases process were raised as important issues. Healthcare professionals need to consider the importance of presenting varied formats of education based on the experiences and preferences of patients with SpA.

## Background

In patients with different chronic diseases such as diabetes, asthma, and arthritis, patient education is a crucial non-pharmacological treatment to better manage the disease and optimise health [1, 2]. Patient education can be described as an interactive process between patients and the healthcare providers [3, 4], through different learning activities, with the aim of supporting and strengthening the patient’s self-efficacy and adherence to both pharmacological and non-pharmacological treatment [2]. The progress in the last decade, from the healthcare provider being seen as the expert, towards an active process between the healthcare provider and the patient, is well accepted. This principle of shared decision making between the healthcare provider and the patient is a cornerstone in the management of patients with different chronic diseases, where the patient takes an active role in decision making regarding his or her health [3, 5, 6]. Patient education can be given as a group intervention or as an individual intervention on a one-to-one basis, in different settings [1, 7, 8]. The patient education includes both information on the disease – for example, concerning symptoms, consequences, treatment, physical activity, exercise, and assisting devices – and emotional support, such as the confidence to act (self-management) and discussion of influences on social circumstances [1, 3]. Individually tailored education have been requested [7] but often the prioritisation of topics to be discussed still differs between patients and health professionals [5]. Patient education has spread more widely than within healthcare itself, with e-health and web-based health resources such as telehealth platforms, online communication services, and smartphones emerging as new methods of communication and education [3, 9–11].

Spondyloarthritis (SpA) is a rheumatic umbrella diagnosis covering different sub-diagnoses [12, 13]. SpA is chronic in most cases, with the main physical symptoms in the axial skeleton. The patient often has an early disease onset and a life-long burden affecting several aspects of life – physical, mental, and work-related – as well as the effects of the disease on health-related quality of life [14–19]. Different experts in the field of arthritis have recommended a combination of pharmacological therapy and non-pharmacological treatment – including regular exercise and patient education – as an integral part of the disease management of SpA [2, 20–23]. Evidence based recommendations for education in patients with arthritis in general have recently been developed, addressing when, how, to whom, and by whom the education should be delivered and evaluated [2].

There have been widely different approaches to the structure of educational interventions, with effects on self-efficacy, psychological functioning, and physical functioning, even though all interventions are not necessarily equally suited to all patients with arthritis [24–31]. There is a demand for flexible management [26], and patient education in patients with arthritis should be individually tailored to the patient’s specific needs and priorities [2, 8, 32]. Most of the research in the field of arthritis is based on patients with rheumatoid arthritis, which is the most common rheumatic disease. The form of patient education methods that is best suited to patients with SpA with an early disease onset has seldom been studied, but it is of interest, as it applies to a different―that is, younger―target group [2, 21]. Earlier research in patients with SpA has produced some divergent results concerning satisfaction with patient education, where women have expressed more needs for education than men [32–34]. Recent results on patients with SpA have shown that diagnosis, prognosis, and management are the most important educational topics requested by patients, and in qualitative analysis medication, pain, fatigue, activity, work, and prognosis were raised as important issues [35].

The aim of the present study was twofold. First, to identify the needs and priorities in patient education in patients with spondyloarthritis, based on gender, disease duration, and self-reported disease activity. Second, to investigate patients’ experiences and preferences when receiving patient education.

## Methods

In a mixed-methods design, the convergent parallel method was used [36, 37] to gain better insight into different aspects of the educational needs of patients with SpA. The quantitative part of the study was based on data from a cross-sectional postal questionnaire survey in 2012. The qualitative part of the study was conducted with patients from the cohort, as two focus groups with in total six patients and as five individual interviews between May 2013 and May 2015. This part had a descriptive design with a qualitative manifest conventional content-analysis approach [38].

### Subjects

The study population of 420 patients with SpA was randomly chosen from a specialist rheumatology clinic register at a hospital for patients with rheumatic diseases in the southwest region of Sweden. Half of the study population, 210 subjects, had ankylosing spondylitis (International Classification of Diseases tenth revision, (ICD-10) diagnosis M45) and the other 210 subjects had undifferentiated spondyloarthritis (ICD-10 diagnosis M46.0, M46.1, M46.8, and M46.9), both of which are sub-diagnoses. All patients were given the diagnose at least once at the rheumatology clinic. The eleven patients who were interviewed were recruited in order to achieve variations in experiences and strategic sampling was used in terms of age, sub-diagnosis, gender, and self-reported disease duration, disease activity, physical function, and quality of life (Table 1).

**Table 1:** Characteristics of the 11 patients with spondyloarthritis who were interviewed

Characteristics	Patients
Age, years, min–max	38–66
Gender	7 men, 4 women
Sub-diagnoses	5 with AS, 6 with USpA
Disease duration, years, min–max	8–48
Education, years	10–19
BASDAI, 0–10, min–max	1–6
BASFI, 0–10, min–max	0–5
EQ5D, min–max	0.29–1.0

*AS* ankylosing spondylitis, *USpA* undifferentiated spondyloarthritis, *BASDAI* Bath Ankylosing Spondylitis Disease Activity Index, *BASFI* Bath Ankylosing Spondylitis Functional Index, range 0 (worst) to 10 (best), *EQ5D* EuroQol 5-domains, range 0 (worst) to 1 (best)

### Procedures

#### Questionnaire survey

The questionnaire contained three different patient-reported and disease-specific instruments; the Arthritis Educational Needs Assessment Tool (ENAT) [39, 40], the Bath Ankylosing Spondylitis Disease Activity Index (BASDAI) [41], and the Bath Ankylosing Spondylitis Functional Index (BASFI) [42] together with self-reports of disease duration and level of education. Patient characteristics such as age, gender, and sub-diagnosis were collected from the patient register at the rheumatology clinic.

The ENAT is a self-reported outcome measure assessing patients’ educational needs, and it has been validated for different rheumatic diseases [39, 40]. The questionnaire has been translated into Swedish and six other European languages, and is available as a valid and reliable instrument for cross-culture comparisons [4, 40, 43]. The ENAT starts with some initial questions concerning descriptives (age, gender, disease duration, educational level). Two general questions follow, concerning whether they currently have a need for patient education (yes/no) and to what degree (5-point Likert scale). The next part of the questionnaire consists of 39 items falling into seven different domains, which measure specific aspects of educational needs. Each item can be rated as being not at all important (score 0) to extremely important (score 4) on a 5-point Likert scale. The different domains include managing pain (score 0–24), movements (0–20), feelings (0–16), the disease process (0–28), treatment from health professionals (0–28), self-help (0–24), and support from others (0–16), with a total score ranging from 0 to 156 points (and with higher scores indicating greater needs) [39, 40].

The BASDAI and the BASFI are two disease-specific patient-reported outcome measures. The BASDAI [41], measuring disease activity, consists of six questions on pain and stiffness, and the BASFI [42], measuring physical function, has ten questions. The final scores are estimated by calculating the mean scores with a total score ranging from 0 (best) to 10 (worse).

#### Interviews

Before the interviews took place, the patients were contacted with a postal invitation letter. A telephone call followed, and the patient and interviewer made an arrangement about the time and location of the interview. The two focus groups and two of the individual interviews took place in a quiet room at the research centre. Another interview was conducted in a place chosen by the patient in connection with his work, and the last two were conducted by telephone. All interviews were conducted by the same researcher (EH). At the focus group interviews, there was also an observer (IL) who took notes and contributed with additional follow-up questions.

In connection with the interviews, the BASDAI, the BASFI questionnaires were also administered together with the health-related quality of life EuroQol-5 Domain (EQ-5D) [44] which is a generic questionnaire. The EQ-5D has a summary score ranging from 0 (no health) to 1 (full health).

The focus groups and the interviews lasted between 30 and 60 min, and were carried out as a conversation based on three opening phrases about experiences of and preferences for patient education, thus ensuring that the interviews followed a similar pattern (Table 2) [45]. Follow-up questions were adapted based on each situation, but were generally intended to make the patients explain their experience further. Two of the individual interviews were pilot interviews conducted in order to test the relevance of the opening phrases, and these interviews were included in the analysis, as no adjustment was found necessary.

**Table 2:** Opening phrases used in the eleven interviews

Opening phrases	
Describe your experiences of the patient education you have received	
What educational needs are important to better manage the consequences of the disease for your everyday life?	
How do you prefer to receive patient education?	

### Data analysis

#### Quantitative analysis

All the quantitative analyses were performed using SPSS software, version 20 for Windows. The descriptive data are presented as mean with standard deviation (SD) or as frequencies (%). The ENAT domain score is commonly also presented as “% of the domain total score”. The raw ENAT scores were converted by using Rasch-transformed values, cross-culturally validated for the specific diseases [4, 46]. The chi-square test was used to make comparisons between group frequencies. The independent-samples t-test was used to make group comparisons of mean domain scores between men/women, disease duration (≤/> 10 years) and disease activity (BASDAI score (≤/> 4) *P*-values of <0.05 were considered to be statistically significant.

#### Qualitative conventional content analysis

A manifest qualitative conventional content analysis [38] was done, showing the visible components of the text, aiming to describe and explore SpA patients’ educational experiences regarding their needs and in what way they preferred to receive information. All focus group interviews and the individual interviews were recorded, and then transcribed verbatim by a secretary. The analysis was to remain close to the text and preserve contextual meanings, and to continually switch between the whole of the text and parts of it. The entire text from all the narratives was read through several times, and sentences or phrases containing relevant information were identified―resulting in 202 meaning units. These were summarized to condensed meaning units, in order to shorten the text but with the intension of preserving the content. In the next stage, the condensed meaning units were abstracted and provided with codes. Codes with similar meaning were grouped into three categories reflecting the central message, i.e. the manifest content of the interviews, providing knowledge and understanding of educational experiences. The analysis was done by two different researchers (EH and IL), first independently, later the analysis was compared and discussed until consensus was reached.

## Results

### Needs and priorities concerning patient education

A total of 183 patients (46%) with SpA responded to the postal survey and were included in the quantitative data analyses. Patients with AS were more prone to respond to the questionnaire survey (50%) compared to patients with USpA (37%), *p* < 0.01. There were no differences between responders and non-responders concerning age (*p* = 0.21) and gender (*p* = 0.12).

The age of the responders ranged from 21 to 85 years, and the mean age was 53 years (SD 13). Data on the demographics and characteristics of the patients with SpA stratified by gender and sub-diagnosis are given in Table 3.

**Table 3:** Characteristics of the patients with spondyloarthritis who responded to the questionnaire survey

	SpA Total	SpA Men	SpA Women	AS	USpA
	*n* = 183	*n* = 100	*n* = 83	*n* = 106	*n* = 77
Age, years, mean (SD)	53 (13)	55 (14)	51 (12)	55 (14)	50 (12)
Gender, % men	55%	-	-	67%	38%
Disease duration, years, mean (SD)	21 (13)	24 (13)	17 (10)	25 (13)	16 (11)
BASDAI, 0–10, mean (SD)	4.7 (2.1)	4.3 (2.1)	5.1 (2.1)	4.6 (2.3)	4.9 (1.9)
BASFI, 0–10, mean (SD)	3.4 (2.4)	3.3 (2.3)	3.6 (2.4)	3.6 (2.4)	3.1 (2.3)
Education, years, mean (SD)	12 (3)	12 (3)	13 (3)	12 (3)	13 (3)

Presented in total, according to gender, and according to sub-diagnoses *AS* ankylosing spondylitis, *USpA* undifferentiated spondyloarthritis, *SD* standard deviation, *BASDAI* Bath Ankylosing Spondylitis Disease Activity Index, range 0 (worst) to 10 (best), *BASFI* Bath Ankylosing Spondylitis Functional Index, range 0 (worst) to 10 (best)

Four out of ten patients with SpA (43%) reported having educational needs to better manage their disease by answering yes to the first question. Patients with high disease activity more often reported educational needs than patients with low disease activity (54 vs. 23%; *p* < 0.01). There was a trend for patients with a longer disease duration (>10 years) to more often report educational needs than patients with a disease duration of 10 years or less (46, vs. 29%; *p* = 0.06). No significant differences were found of those reporting educational needs (yes/no) in men and women with SpA (37 vs. 47%; *p* = 0.11), or between the different subgroups AS and USpA (44 vs. 41%; *p* = 0.62).

The highest percentages of educational needs were reported in the domains of self-help (67%), feelings (63%), and the disease process (61%) (Table 4).

**Table 4:** Arthritis Educational Needs Assessment Tool (ENAT) score (converted) by domain and in total

Domain	Total sample, *n* = 170, mean (SD)	% of the domain total score^a^	Men, *n* = 92, mean (SD)	Women, *n* = 78, mean (SD)	*p*-value	Disease duration ≤10 years, *n* = 42, mean (SD)	Disease duration >10 years, *n* = 125, mean (SD)	*p*-value	BASDAI ≤4, *n* = 64, mean (SD)	BASDAI >4, *n* = 116, mean (SD)	*p*-value
Pain (0–24)	12 (6)	50%	11 (6)	13 (6)	0.02	12 (7)	12 (6)	0.87	10 (5)	14 (6)	<0.01
Movement (0–20)	11 (5)	55%	10 (5)	12 (5)	<0.01	10 (6)	11 (5)	0.62	8 (5)	13 (5)	<0.01
Feelings (0–16)	10 (4)	63%	9 (4)	10 (4)	0.02	9 (5)	10 (4)	0.71	8 (4)	10 (4)	<0.01
Disease process (0–28)	17 (7)	61%	17 (7)	18 (7)	0.10	16 (8)	17 (6)	0.46	17 (7)	17 (7)	0.64
Treatments (0–28)	14 (7)	50%	15 (7)	14 (8)	0.60	13 (8)	14 (7)	0.61	12 (7)	15 (7)	0.01
Self-help (0–24)	16 (6)	67%	15 (6)	17 (5)	<0.01	16 (6)	15 (5)	0.31	15 (6)	16 (6)	0.12
Support (0–16)	8 (4)	50%	7 (4)	8 (4)	0.17	7 (4)	8 (4)	0.28	6 (4)	9 (4)	<0.01
Total score (0–156)	88 (31)	56%	83 (31)	94 (29)	0.03	84 (34)	88 (29)	0.58	76 (28)	94 (31)	<0.01

Converted scores presented as mean and standard deviation (SD) in total, stratified by gender, disease duration above or below 10 years, and BASDAI score above or below 4, in patients with spondyloarthritis (*n* = 178; 99 men and 79 women). Higher scores indicate higher educational needs. ^a^the domain mean divided by the domain total score

### Differences between groups of domain and ENAT total scores

Women reported greater educational needs in the domains pain (*p* = 0.02), movement (*p* < 0.01), feelings (*p* = 0.02), and self-help (*p* < 0.01). Women also had greater educational needs when analysing the ENAT total scores (mean difference = 10.4, 95% CI: 19.6-1.1, *p* = 0.03) (Table 4).

Patients with a higher disease activity reported higher domain scores in pain, movement, feelings, support (all *p* < 0.01), and treatment (*p* = 0.01). Patients with a higher disease activity also had greater educational needs when analysing the ENAT total scores (mean difference = 17.9, 95% CI: 27.4-8.5, *p* < 0.01) (Table 4). There were no differences in educational needs based on the disease duration, neither for different domains or for ENAT total scores (Table 4).

### Patients’ experiences and preferences when receiving patient education

Patients’ experiences and preferences in patient education emerged in three categories: guiding education, reliable education, and available education.

*Guiding education (GE)* comprised SpA management including education and knowledge concerning the disease, knowledge of physical and mental symptoms, treatment, prevention strategies, and prognosis. The patients had experience of such education, but also the lack of it. They highlighted self-management as an important issue of GE, such as how to reduce pain with or without medication, how and when to exercise, diet, alcohol intake, and how to handle the work situation. GE about the effect of climate, heredity, and assisting devices was also emphasised. The patients had experienced that GE changed over time, depending on the disease duration and disease activity. Quotations are given in Table 5.

**Table 5:** The three categories with quotations describing the patients’ experiences and preferences concerning patient education

Categories	Quotation
Guiding education	*“It is probably mostly the consequences that you want to know more about and what to do to feel healthier”.* “*What you can do by yourself, information concerning medicines and treatments, and tips on where to go. You also want to know if there is something you shouldn’t do”.*
Reliable education	*“Advice and information should come from people who work with it, people who know what they’re talking about.”* *“To read about others in the same situation gives me important information.”* “*It’s important that it is tailored to me.*” *“I am actively looking for webpages that I know are reliable and robust”* *“If you have your instructor there over the internet, who sees exactly what you do, of course you will feel more secure”.* *“I don’t trust online information that I have searched for myself, and don’t know if the information is right or wrong ― or whether it is applicable to me.”*
Available education	*“I prefer to receive the education orally. Written education could also work, but it would be better to discuss and to talk.”* *“I get information from my personal experiences, as I have come to know the symptoms”.* *“If I had been able to use Skype and FaceTime, the professional could have provided comments if I, for example, did exercises in the correct or wrong way.”* *“I think it’s good to be able to absorb the education when you have the time and want to have it. Then you are more receptive to the information given.”* *“This thing with blogs is pretty good.”*

*Reliable education (RE)* involved how and by whom the patient education was communicated. There were experiences of RE based on science, based on the patient’s own experience, communicated by specialists, or communicated by patient with long-term experience of the disease, but experiences of other patients could also be perceived as being less reliable. The patients also described experiences of RE through resources such as the Health Care Guide (giving information on health and healthcare), the National Health Service website, the Swedish Rheumatism Association’s homepage, and less reliable education given in blogs and magazines*.* Skewed information or a large amount of patient education from various sources was perceived as being less reliable. Concerning the future, they described reliable education in terms of getting feedback over the internet, for example with regard to proper exercise (Table 5).

*Available education (AE)* involved patients’ experience of the education to be presented in various formats. AE in terms of individual contacts with healthcare professionals when required was put forward as being important. The AE could be available orally through meetings, phones, lectures, videos, rehabilitation visits, or as nicely presented written information in pamphlets, e-mails, magazines, links, webpages, and apps, or as scientific papers. The available education was also obtained through patients’ own personal experiences*.* The patients believed that the education should be accessible when their questions are raised**,** the information should be available in a varied manner regardless of the time of day or night. New methods of AE were requested (e.g. Skype, video, chat forums, and FaceTime), and the use of e-mail communication. Some patients described less good experiences when using the internet and chat forums, for example, not knowing what information was relevant. Quotations are given in Table 5.

## Discussion and conclusion

Patient education is an important and recommended non-pharmacological treatment for patients with different chronic diseases, including SpA [22, 23]. Earlier studies in the field have concentrated on patients with rheumatoid arthritis, and not much research has been done in patients with SpA [11, 31]. The results from the present study showed that almost half of the patients with SpA considered that they had a need for education, and the areas concerning self-help, feelings, and the disease process were those that were most requested. The same areas were also highlighted through the categories that emerged from the qualitative data; the importance of receiving patient education that was guiding, reliable, and available in order to manage the consequences of the disease better. Individualized and tailored education was requested but also newer methods such as Skype, video, and chat were mentioned as possible resources, even with regard to getting trustworthy feedback–for example, during exercise. However, online sources were also mentioned as being less reliable resources. Increased collaboration between healthcare professionals, patient associations, and pharmaceutical companies could verify that the information is reliable and constantly updated.

### Proportion of patients with educational needs

In the present study, we found that nearly half of the patients with SpA reported having educational needs. In a recent study of patients with different inflammatory diseases (rheumatoid arthritis, ankylosing spondylitis, and psoriatic arthritis), 60% expressed the need for more information about medication, diagnosis, and exercising [34], which was a larger proportion than in the findings of the present study. One explanation for this could be the association between educational needs and disease activity [32]. Giacomelli et al., used a convenience sample of patients with high scores in self-reported disease activity [34] while the present study randomly selected patients from a rheumatology clinic register, without confirmed current health care needs. However, in the present study the proportion of patients with educational needs were significantly higher in the group of patients with BASDAI scores of more than 4.

### Domains of importance

The three most important domains mentioned by the patients included in the questionnaire survey were self-help, feelings, and the disease process. These issues were also mentioned as being of importance in the interviews, and fell into the “guiding education” category in the qualitative analysis. Radford et al. found educational needs about symptoms and support related to emotional needs as being of importance when tailoring patient education [7]. Other studies have identified similar domains of importance, where educational needs concerning diagnosis, prognosis, medication, exercise, and activities were reported [34, 35]. In a study by Kjeken et al., the patients most often asked for information concerning the disease (diagnosis, medications), exercise, and activities, irrespective of the inflammatory diagnosis [26]. Thus, it does not appear to matter which underlying rheumatic disease, or even chronic disease, the patient has when it comes to patient education, while how to tailor the education to the individual patient is probably more important [26, 47].

### Differences between groups

Cooksey et al. found that women had more educational needs than men in the domains self-help, pain, and movement, which is in accordance with the results of the present study. Women have also by others been reported to gather more health information than men [33]. Also in other self-reported health variables, women and men have been found to have a different way of reporting health [48] which may have had an impact on the present results. Also patients with BASDAI scores above 4, had higher needs in several domains, also reported by others [32, 34]. However, based on the results of this study, disease duration does not appear to affect the demand for patient education.

### New sources of communication

Websites, online audio/video, and e-learning have already been suggested to be useful sources of information [2, 35]. Some studies have shown differences between the genders concerning use of the internet, but through the years such differences appear to have diminished [49]. Our qualitative data strengthen the fact that patients with SpA were also interested in new resources (Skype, video, chat forums, FaceTime, and e-mail), but some also told of less good experiences with online information and blogs. The health profession must form and maintain good internet forums with reliable information. The use of e-health and internet as a reliable resource to deliver patient education is something to work on. In order to better meet the patients’ needs it could be essential for the healthcare professionals to closely collaborate with other stakeholders in delivering reliable information. For example, healthcare professionals, patients associations, and possibly pharmaceutical companies could merge together to verify that the information given at different internet forums is relevant and reliable.

### Limitations and methodological considerations

One limitation of the present study is that the results may not be applicable to the youngest patients with SpA. Even though the inclusion criteria included a wide range of disease duration, the tables show that there was a high mean duration of disease and high mean age in both the quantitative and the qualitative parts. Another limitation of the study was the low response rate which may have introduced response bias, thus affecting the generalizability. The ENAT questionnaire has also limitations due to the seven fixed domains. These domains do not cover all information of importance in patients with SpA. In order to compensate for this limitation, the interview questions were held open and thus, the mixed-method design creates greater credibility of the patients’ priorities in the current study.

In the qualitative part, aspects of credibility, dependability, confirmability, and transferability were taken into consideration [50]. Although relatively few interviews were conducted, a sufficient number of meaningful units were revealed, and the sample size is considered to be less important in qualitative research. Even so, the interviews only reflect the experiences of a small patient sample, and the results can be used mainly to suggest important topics for future work. The dependability of the data was strengthened by using the same opening questions and the same interviewer (EH). The interviews were held in an undisturbed environment by a researcher familiar with the topic. Furthermore, the interviews were repeatedly and systematically read, analysed, and interpreted by two researchers, which strengthened the confirmability and the topic was easy to talk about, which strengthened the transferability [50].

To gain trustworthiness, all researchers were working with the different parts of the study. All the researchers had a long experience of the patient group, of epidemiological research, and of qualitative research. They had different professional experience, which widened the standpoint of the analyses and conclusions.

#### Conclusion

There is a demand for patient education, where almost half of the patients with SpA expressed a need for more. Self-help, feelings, and diseases process were raised as being important domains of patient education. The qualitative data further supported the existence of these domains and highlighted the importance of obtaining a patient education that was guiding, reliable, and available, to make life easier when living with the disease. These needs fit in well with the reports of needs in other patient groups with different chronic diseases.

#### Practical implications

Healthcare professionals must consider the importance of presenting individually tailored and varied formats of patient education based on patients’ experiences and preferences, and making better use of other possible sources. Meetings with various specialists, opportunities to have contact with other patients, and newer ways of communicating education appear to be good ways of helping the patients to better manage their SpA. In each unique meeting with a patient with a chronic inflammatory disease, a professional with knowledge of the individual’s needs is required. The present study indicates that there is more than one solution regarding the content and structure of patient education for patients with chronic inflammatory diseases. The use of a simple screening tool, such as the ENAT, in clinical practice to obtain a basic knowledge of the patients’ needs may be a starting point for further exchange of knowledge.

In future research, the short-term and long-term effects of individualized and tailored patient education, based on these needs, should be evaluated in terms of both health outcomes and cost-effectiveness in this group of patients.

## Abbreviations

AE: Available education
AS: Ankylosing spondylitis
BASDAI: Bath ankylosing spondylitis disease activity index
BASFI: Bath ankylosing spondylitis functional index
ENAT: Educational needs assessment tool
EQ5D: EuroQol-5 dimensions
GE: Guiding education
ICD-10: International classification of diseases, tenth revision
RE: Reliable education
SD: Standard deviation
SpA: Spondyloarthritis
USpA: Undifferentiated spondyloarthritis
